# An Implementation Evaluation of A Group-Based Parenting Intervention to Promote Early Childhood Development in Rural Kenya

**DOI:** 10.3389/fpubh.2021.653106

**Published:** 2021-05-05

**Authors:** Jill E. Luoto, Italo Lopez Garcia, Frances E. Aboud, Daisy R. Singla, Rebecca Zhu, Ronald Otieno, Edith Alu

**Affiliations:** ^1^RAND Corporation, Santa Monica, CA, United States; ^2^Department of Psychology, McGill University, Montreal, QC, Canada; ^3^Department of Psychiatry, Sinai Health and University of Toronto, Toronto, ON, Canada; ^4^Department of Psychology, University of California, Berkeley, Berkeley, CA, United States; ^5^Safe Water and AIDS Project (SWAP), Kisumu, Kenya

**Keywords:** parenting intervention, implementation evaluation, rural Kenya, CARE guidelines, early childhood development

## Abstract

Early childhood development (ECD) parenting interventions can improve child developmental outcomes in low-resource settings, but information about their implementation lags far behind evidence of their effectiveness, hindering their generalizability. This study presents results from an implementation evaluation of Msingi Bora (“Good Foundation” in Swahili), a group-based responsive stimulation and nutrition education intervention recently tested in a cluster randomized controlled trial across 60 villages in rural western Kenya. Msingi Bora successfully improved child cognitive, receptive language, and socioemotional outcomes, as well as parenting practices. We conducted a mixed methods implementation evaluation of the Msingi Bora trial between April 2018 and November 2019 following the Consolidated Advice for Reporting ECD implementation research (CARE) guidelines. We collected qualitative and quantitative data on program inputs, outputs, and outcomes, with a view to examining how aspects of the program's implementation, such as program acceptance and delivery fidelity, related to observed program impacts on parents and children. We found that study areas had initially very low levels of familiarity or knowledge of ECD among parents, community delivery agents, and even supervisory staff from our partner non-governmental organization (NGO). We increased training and supervision in response, and provided a structured manual to enable local delivery agents to successfully lead the sessions. There was a high level of parental compliance, with median attendance of 13 out of 16 fortnightly sessions over 8 months. For delivery agents, all measures of delivery performance and fidelity increased with program experience. Older, more knowledable delivery agents were associated with larger impacts on parental stimulation and child outcomes, and delivery agents with higher fidelity scores were also related to improved parenting practices. We conclude that a group-based parenting intervention delivered by local delivery agents can improve multiple child and parent outcomes. An upfront investment in training local trainers and delivery agents, and regular supervision of delivery of a manualized program, appear key to our documented success. Our results represent a promising avenue for scaling similar interventions in low-resource rural settings to serve families in need of ECD programming. This trial is registered at ClinicalTrials.gov, NCT03548558, June 7, 2018. https://clinicaltrials.gov/ct2/show/NCT03548558.

## Introduction

An estimated 43 percent of children under age 5 in LMICs experience compromised cognitive and socioemotional development due to poverty, poor nutrition, and inadequate psychosocial stimulation (1). Early childhood development (ECD) interventions have been shown to improve child development outcomes in low- and middle-income (LMIC) settings among children under 3 years of age (2, 3). Evidence from the most recent Lancet series (1, 4, 5) highlights the critical importance of nurturing care, a term coined to characterize the necessary inputs for ECD, namely good health, adequate nutrition, security and safety, and responsive caregiving with opportunities for early learning. The Nurturing Care Framework (NCF) (6) was created in 2018 by the World Health Organization, UNICEF and the World Bank to recommend the promotion of responsive care, early learning activities, and nutrition support for children under 3 years of age (7). Health Ministers from around the world have pledged to implement the NCF.

Governments and organizations in LMICs are now asking for operational guidance on how to implement effective and scalable programs to improve ECD outcomes. Though there is now consistent evidence of effectiveness of ECD interventions to improve child outcomes, there is strikingly very little information about how these programs were implemented to demonstrate how such results were achieved. There are many ways to implement ECD programs, and decisions must be made regarding: program content, duration and intensity, delivery mode –whether group-based meetings, clinic or home visits– and the necessary qualifications of delivery agents. Some designs may be more suitable for low-resource settings where cost is an issue (8) and where adult literacy and awareness of ECD may be low (4, 9).

To our knowledge, only two published effectiveness studies from Uganda (10) and Pakistan (11) provide information on the process of implementing an ECD parenting program in an LMIC setting that addresses NCF practices. However, they did not identify contextual factors considered when developing or adapting their programs, such as how the needs of parents influenced the program content, and how the capacity of providers influenced the training offered. They also did not examine the determinants and consequences of implementation outputs. Since then, a more structured guideline has been published for reporting implementation of early childhood development programs called the Consolidated Advice for Reporting of ECD implementation research (CARE), (12) along with options for measuring implementation processes (13).

Using the CARE guidelines, this study describes the implementation of a successful parenting intervention that compared the effectiveness of two group-based potentially scalable delivery models for an integrated ECD intervention for families with young children in rural Kenya (see Supplemental Material). Because individual home visits in dispersed rural settings like ours would be prohibitively labor intensive and expensive for scaling, one delivery model featured only group meetings at the village level, while the other combined a small number of home visits with group meetings, as advocated by some (3). The responsive stimulation and nutrition education intervention was named Msingi Bora (“Good Foundation” in Swahili). In collaboration with a Kenyan non-governmental organization (NGO), the Safe Water and AIDS Project (SWAP), we conducted a community-based multi-arm cluster-randomized trial to evaluate the effectiveness of Msingi Bora, implemented across 60 villages in three rural sub-counties using Kenya's network of community health volunteers (CHVs) (14). In 20 intervention villages, CHVs delivered a total of 16 fortnightly group-based sessions within their villages. In another 20 villages, CHVs delivered the same program, but combined 12 group sessions with 4 home visits. A third group of 20 villages served as a comparison arm. Among the 40 villages assigned to an intervention arm, 20 villages (10 from each arm) invited only mothers and children, while the remaining 20 villages also invited fathers to the 16 sessions. On average, each village had 19 eligible children invited to attend sessions with their mothers. Our results showed that parenting interventions delivered by trained para-professional CHVs in large mother-child groups can be effective in promoting child cognitive, language and socio-emotional development, as well as parental stimulation practices in low-resource settings with potential for scalability (14).

**Figure 1:**
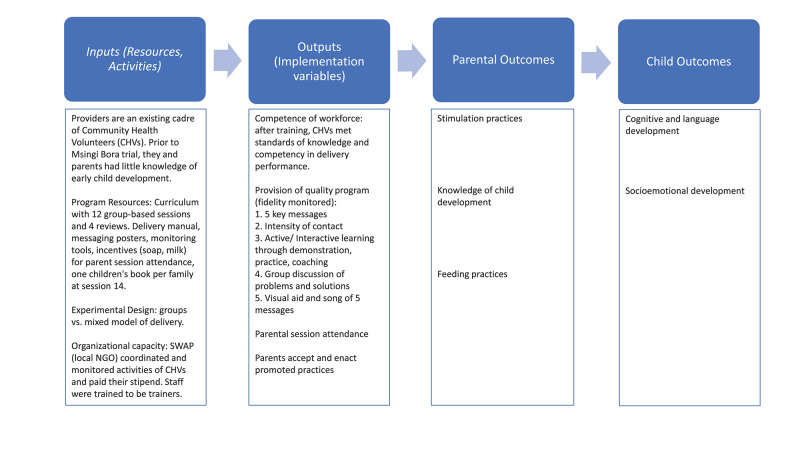
Logic model. Child and parent outcome indicators are reported in Luoto et al. (14).

The current manuscript reports results from an implementation evaluation of the Msingi Bora trial, with a view to examining determinants and consequences of implementation outputs, such as acceptance of the program and fidelity of delivery, and their association with parent and child outcomes at endline (15, 16). Outcomes were reported in the impacts paper but are examined here in relation to implementation indicators. Using a logic model framework (Figure 1), we conducted a mixed methods study to assess Inputs (e.g., workforce and parenting context, program resources such as curriculum, and design), Outputs (e.g., post-training competence of workforce, fidelity of delivery, attendance by parents, adoption by parents), and Immediate Outcomes (e.g., parental behaviors, child outcomes).

Our research questions were:

- What program resources were input ahead of implementation? Was the program content and delivery acceptable to parents and delivery agents?
- How did Outputs such as delivery *fidelity* and parental *attendance* and *enactment (i.e., adoption of promoted practices)* change during the intervention, and what determined these Outputs?
- What features of Inputs and Outputs were, in turn, associated with child and parent Outcomes, and how did this differ by delivery mode?

## Materials and Methods

### Setting and Participants

We conducted a mixed methods implementation evaluation of the Msingi Bora trial between April 2018 and November 2019 in three sub-counties outside Kisumu. These predominantly rural areas are characterized by high rates of poverty, child mortality, stunting (23%), and spousal violence (60%); (17) 56% of children under 2 years of age had at most one plaything, (18) and few parents understand the need for stimulation from birth (19). Spoken languages include Swahili, Luhya, and Luo. The majority of villagers are subsistence farmers or unskilled informal workers. There is no ECD policy pertaining to ages under 3 years in this region.

#### Study Parents

The eligibility criteria for parent participants within villages were mothers or other female primary caregivers aged 15 and over with a child between 6 and 24 months without signs of severe mental or physical impairment. Baseline demographic data from 1,152 households across 60 villages are available in Luoto et al. (14).

#### Delivery Agents

Program delivery agents were existing village Community Health Volunteers (CHVs), which is a part-time and voluntary position under the Ministry of Health (MOH) tasked with improving community health through home visits (20).

#### Supervisors

We worked with three Kisumu-based staff and seven sub-county supervisors (4 males, 6 females) from the local NGO to oversee the daily operations of Msingi Bora and to provide training and supervision to delivery agents.

#### Ethical Approvals

All mothers provided written informed consent at the time of data collection. Selected CHVs and SWAP supervisors provided consent to be interviewed at the end of the intervention. Ethics approval was obtained from Maseno University in Kisumu, Kenya, and RAND. The trial is registered at ClinicalTrials.gov, NCT03548558, and the study protocol has been published (21).

### Implementation Strategy

The implementation evaluation was guided by the CARE guidelines and organized by the logic model in Figure 1.

### Content and Delivery Strategy

Msingi Bora's parenting intervention was based on a structured curriculum adapted from previous successful parenting trials in LMICs, and expanded to include more activities around responsive play and talk with children (22, 23). Documented evidence on low awareness about ECD among parents and CHVs (18, 19) informed the need for a structured and manualized set of activities for each session, along with time for guided discussions among parents.

Six sessions were piloted in May-June 2018 in six villages not included in the main trial. The pilot confirmed the need for a structured manual of activities and that CHVs were not familiar with hosting group sessions on ECD. It also helped us recognize the need to reduce lengthy speeches by the CHV in favor of practical activities, and to provide a streamlined manual that could be easily navigated by CHVs. The main trial tested group-only delivery against a mixed model combining group sessions with home visits, but in both arms group sessions were the main format for delivery of messages (8).

The finalized curriculum included a total of 16 fortnightly sessions with session-specific activities and materials, and Luo or Swahili and English manuals for each CHV. The structured curriculum was based on five “key” practices: responsive play, responsive communication, hygiene, nutrition, and love and respect (22). The Responsive Play and Communication curriculum was informed by research on responsive stimulation (24). The Love and Respect curriculum used a range of culturally-adapted interpersonal, cognitive and behavioral treatment elements as the basis for promoting maternal well-being and healthy family dynamics (22, 25). Every fourth session served as a review session, which aimed at consolidating knowledge and new practices learned in the preceding sessions. For these sessions, households in the group-only arm continued with group meetings while households in the mixed-delivery arm received individual home visits from their CHV during the same week that group review sessions were held. During these home visits, CHVs delivered identical review messages to those in the group reviews, but the focus was personalized on that family.

Mothers and children were invited to attend every session. All sessions emphasized parents learning new practices with their child or spouse through demonstration and coached practice, group-based problem solving and peer support, along with homework to play and talk with their child.

A poster with each of the five practices illustrated was used in every session. To help families recall the practices, they received a small version of the poster to take home after the first session. An illustrated Kenyan story book in Luo or Swahili was given to each child in Session 14, to be used during following sessions and at home to encourage responsive communication. For the seven responsive play and communication sessions, parents were taught how to play games using materials available in homes and adapt the games to make them more challenging as the child aged. Parents were not given toys or playthings, because they would be costly and not appropriate as the child aged. Instead, they were encouraged to have a playbag with play objects that can be found at home such as bottle caps, ball, sticks, colored cloths, and pictures. Parents were reminded to add things monthly. CHVs also collected materials for a play bag and brought this to sessions.

Mothers received a small incentive for timely attendance, namely a small bar of soap, based on piloting. In later sessions, as all children were above 12 months, small pouches of milk were distributed instead of soap (each valued at USD $0.15). Sessions took place in local community centers or churches. Households in villages assigned to the comparison group did not receive any interventions besides information about child feeding during the baseline survey.

### Delivery Personnel

CHVs are members of their own communities and the position is generally part-time. Minimum qualifications include being literate, permanent residents of the village, and accepted in their roles by the community members. Upon selection, CHVs undergo roughly 10 days of training held by county MOHs on topics related to basic health promotion; (20) ECD topics are not addressed. For our study, CHVs in villages randomly assigned to an intervention arm were invited to deliver the program; none refused. The research project paid CHVs a monthly stipend of USD $20 for their duties according to local policy.

CHVs were trained on Msingi Bora in two parts. Training of sessions 1–8 took place at the start of the program over 8 days; training of sessions 9–16 took place at midline over 8 days. The first training was conducted in English by international researchers (JL, IL, FA, and DS) with all CHVs together, though they had an English and local language version of the manual. The second training was conducted in the local languages by SWAP staff following a train-the-trainers model in which international staff first trained SWAP staff, who then trained the CHVs. Beginning with session 4, monthly 1-day refresher trainings were also performed in each sub-county to help CHVs prepare ahead of each session.

Training focused on three critical skills: (a) knowing what children need to develop; (b) demonstrating and coaching caregivers as they engaged in the new practices; and (c) facilitating group discussions about problems and solutions. Trainings followed the sequence of activities in the manual. They emphasized practicing how to demonstrate the activities, coach parents and facilitate group discussions, first among themselves and then with groups of mothers and children from villages not participating in the study.

CHVs underwent tests on knowledge and competency to deliver sessions at the end of each training, the former with a paper-and-pencil test and the latter with mock sessions scored using a fidelity rating sheet (described below). Those who were weaker at the end of training were required to attend the session of a peer and undergo extra practice before delivering their own group session.

To obtain information from CHVs concerning their perspectives of the program content and delivery format, as well as on their training, supervision, and curriculum acceptance, semi-structured interviews were conducted with eleven randomly selected CHVs at the end of the program. Two local researchers individually interviewed selected CHVs, male and female, from different sites in a private, neutral community setting.

### Supervisory Personnel

SWAP (www.swapkenya.org) is a local NGO that has operated since 2005 in western Kenya and served as the local implementing partner overseeing the training and supervision of the CHVs. Since topics of stimulation and responsive parenting were new to SWAP staff, the pilot training was delivered by international researchers in English, and attended by six SWAP staff and six CHVs from the selected pilot villages who were trained to deliver the pilot program. For the full trial, four of the six pilot CHVs were promoted to “mentor” supervisory roles to help with local monitoring of the sessions. As mentioned above, SWAP staff and mentor CHVs participated in the two training courses for the main trial, and led the second one under a train-the-trainers model.

Local SWAP staff and mentor CHVs supervised CHVs in the field. This entailed observing and monitoring each group session and a portion of home visits from each review session in assigned villages using a checklist (see section Fidelity). After the session, they asked CHVs to complete their own self-evaluation form and then provided feedback on their performance.

The same local qualitative researchers conducted two focus groups at the end of the program with SWAP staff – one with the three lead supervisory staff, and a second with three sub-county staff. They were asked the same questions and probed about four key components of the program: (a) acceptance of the program content and delivery; (b) helpful and unhelpful aspects of training and supervising CHVs; (c) how easy and difficult it was for CHVs to demonstrate practices and to facilitate discussion; and (d) benefits and problems associated with group sessions and home visits.

### Fidelity

SWAP supervisors utilized a checklist of fidelity items during sessions and provided CHV delivery agents with feedback immediately after each session. In addition, CHVs completed self-evaluation forms following each session and attendance sheets of parent participants. The supervisor and self-evaluation forms were similar in content, and included 5-point scales, from “poor” (1) to “excellent” (5), to rate 13 dimensions of the CHV's fidelity to session content, demonstration of activities, facilitation of group discussions, coaching, use of visual aids and play materials, and other delivery qualities. At the end of the forms, respondents were asked to rate the overall session quality on a 1–10 scale, as well as how much “fun” participants had on a 1–10 scale to capture the level of engagement by participants.

### Caregiver Attendance, Acceptance, and Enactment of Practices

Qualitative interviews of eight pairs of participant mothers from the three sub-counties were conducted at the end of the program, in a private neutral setting. Mothers were interviewed in pairs in order to collect more information and to make them feel more comfortable by being in the presence of a familiar person. They were selected by SWAP staff to meet criteria of representing each of the different delivery formats (group-only meetings, mixed group-home meetings, with and without fathers invited in their villages), and we requested that they include mothers with both high and low attendance in the program. The same two independent qualitative researchers used a semi-structured guide of questions. The questions focused on participants' attendance and acceptance of the program, enactment or adoption of the practices promoted during sessions and how easy and difficult practices were to enact; they also evaluated the group sessions and home visits.

### Data Collection and Analysis Plan

Table 1 summarizes our qualitative and quantitative data sources and how they were analyzed to answer the three research questions.

**Table 1:** Research questions and data sources crosswalk and analysis plan.

**Research Question**	**Quantitative**	**Qualitative**
1. What **program resources** such as curriculum, design and adaptation were put in place ahead of implementation to fit the needs of parents and delivery agents? Was the program content and delivery acceptable to them?	**Source:** - Household Survey: parental stimulation (FCI) and nutrition practices.	**Source:** - Semi-structured interviews with parents, CHVs, and SWAP staff **Content analysis:** - Need for and acceptability of the curriculum content and delivery format
2. How did Outputs such as delivery *fidelity* and parental *attendance and enactment* change over the 8-month course of the intervention, and what determined these Outputs?	**Sources:** - CHV knowledge test after training - CHV Survey: age, education, experience - Supervisor monitoring forms - CHV self-assessment forms - Parental session attendance records **Analyses:** - Link self- and supervisor monitoring forms and parent attendance data with CHV and household characteristics to explore determinants in multivariate analyses	**Sources** - Semi-structured interviews with CHVs, parents and SWAP staff **Content analysis**: - CHV and SWAP staff perspectives on quality of CHV training, supervision, refresher trainings, and delivery, how easy/hard to demonstrate and coach parents - Parent and CHV perspectives on parents' enactment of new practices and attendance
3. What features of Inputs and Outputs were in turn associated with child and parent Outcomes, and how did this differ by delivery mode?	**Sources:** - Experimental design: random assignment to group-only or group-home sessions. - Endline survey data on parent and child Outcomes - CHV knowledge test after training - CHV Survey: age, education, experience - Supervisor monitoring forms **Analyses:** - Link final Outcome data with experimental interventions, household characteristics, as well as CHV performance and characteristics to explore determinants in multivariate analyses	**Sources:** - Semi-structured interviews with parents, CHVs, and SWAP staff. **Content analysis**: - Benefits of group vs. home sessions, views on program success.

All qualitative implementation data were collected by a team of two local independent researchers, one male and one female, trained by FA and JL. All interviews obtained informed consent prior to beginning, were conducted in the local language of choice, and were audiorecorded, translated to English and transcribed. Interviewers probed for elaborations. Transcriptions and translations were verified between the two independent researchers.

Content analysis was performed to analyse all qualitative data using a pre-arranged list of codes on desired information. For the CHV interviews, two co-authors (FA and RZ) independently coded four interviews after agreeing on a coding scheme and found high inter-rater reliabilities (kappa 0.774, 95% CI 0.68–0.87, z = 13.71, *p* < 0.0001). For the parent interviews, the same two researchers independently coded four after agreeing on a coding scheme (kappa 0.766, 95% CI 0.68–0.85, z = 15.76, *p* < 0.0001). Because there were only two supervisor FGD, two researchers (FA and DS) together coded their responses.

Quantitative data were collected using SurveyCTO on Android tablets. All supervisory staff were supplied tablets and filled out the session monitoring forms directly. Following each session, CHVs completed attendance and self-evaluation forms on paper, and the supervisor photographed the paper forms and transfered them to the tablet. A US-based research assistant cross-checked a random subset of photographed paper forms with SurveyCTO forms periodically to ensure accuracy and inquired if there were any inconsistencies to resolve them.

Final outcomes were collected at baseline and endline by a team of trained local enumerators using SurveyCTO on Android tablets. Child cognitive and language outcomes were measured with the Bayley Scales of Infant Development third edition (Bayley III), (26) and socioemotional development was measured with the Wolke Scale (27). Parent outcomes included stimulation practices measured with the Family Care Indicators (FCI) (28) at baseline, and the more comprehensive Home Observation for Measurement of the Environment (HOME) at endline (29). More details on outcome measures listed in Figure 1 are provided in the impact manuscript (14).

Quantitative analyses used multivariate linear regressions by estimating ordinary least squares (OLS) models for continuous outcomes as a function of program inputs or outputs, as appropriate, and combining different data sources including CHV self-evaluation forms, supervisor monitoring forms, maternal attendance records, and household survey data. More details can be found in Table 1 or in notes to the individual tables in Results. For many analyses we used a composite single index of overall delivery quality that we estimated using Principal Component Analysis (PCA) models on the 13 distinct aspects of delivery rated by supervisors on a 1–5 scale. Similarly, when we relate program inputs and outputs to final project outcomes to address our third research question, we estimated a single index of child development using PCA models on four different child development outcomes measured at endline including cognitive, receptive language, expressive language, and socioemotional outcomes.

## Results

### Question 1

What program resources were input ahead of implementation? Was the program content and delivery acceptable to parents and delivery agents?

Table 2 contains illustrative quotes from a content analysis of qualitative interviews with supervisors, CHVs, and mothers on program adaptations and delivery. All three groups of participants indicated that initially they knew very little about child development and the need for stimulating play and communication, consistent with our expectations. Similarly, low baseline scores on the FCI showed that children had on average 1.4 out of 6 playthings and parents performed 3.3 out of 6 stimulating activities with them. They also had low dietary diversity scores (3.1 out of 7 food categories consumed in past 24 h). Thus, there was a need for messages about playing and talking with the child, and including fish and eggs in their diet. The interviews also made clear that the program's five practices were acceptable to implementers and parents, and that the use of a structured manual with practical activities for CHVs and parents to follow was appreciated.

**Table 2:** Illustrative participant responses regarding need for and acceptance of the designed program.

**Supervisors (2 FGDs)**	**Community Health Volunteers (*n =* 11)**	**Mothers (*n =* 8 pairs)**
**Initially low levels of knowledge and practices in responsive play and talk**		
“This was the first time that children were being provided with playing materials.” [2] “I noted mothers were not really having that initiative of play and talk with the child, they didn't see the need of playing with the child.” [2] “At the beginning, when the CHVs were introduced to the games, even for them to understand the concept really took time. But when they got the idea, then it became a little bit easier.” [1]	“Mothers did not know the importance of play, even in session two. They started seeing the sense when we started session three.” [A] “They would say that they thought it was only the child who was supposed to play but they are mature people they can't play.” [C] “Mothers never knew that you can talk to a baby who has not yet started talking.” [C]	“I grew up believing that children are not supposed to eat eggs because that is what our parents told us. But here we were told that eggs are very important to the child's growth.” [B] “In a day I must play with the child but you see I was not used to that because I never did that with my previous children.” [B] “Even when you are at home making the dolls, someone would come and make fun of you, ‘*What are you doing? Are you also a child?*’” [C]
**Acceptability of program's key messages about play, talk, love & respect, food, hygiene**		
“So the mother was not supposed to read the book from page to page but can tell the story depending on what child can be able to identify and that was great.” [1] “And especially in the- when you are carrying out the idea of games, you start from what the child knows, what the child can do easily.” [1] “Attending the sessions, mothers got to understand the importance of playing with their children and they would actually create time to play with their children and each time you would see an improvement on the play bag and play material” [2]	“Their children were empty [passive], but when they see the progress their children are making they decide to come to know what is next.” [B] “They learned through the pictures which was very helpful because when a child can't talk and you show him pictures, he will nod his head and it will make the child happy.” [C] “Most of them came to see the importance of the play bag. At first they were not able to know its value, they were saying we are not made just to pick anything on the roadside.” [C]	“I was asking myself ‘how can I leave my chores to go and play with the child?’ but I got used to it and I found that it was something very easy.” [A] “At first I did not like the teachings because I thought it was a waste of time. (Laughing) … I came to like later [after a few sessions].” [C] “The play bag encourages me to practice the games. Even if I am busy I will tell the child to go and get the play bag and play.” [C] “When you go at the trainings you will get very good teachings and all the time, we would be shown new things.” [A] “I liked the topic of love. It made me learn a lot in my marriage life and also how to love my children.” [B]
**Acceptability of delivery format: structured, prescriptive set of practical activities—Manual with visual job aides**		
“When we were reviewing these games, I felt happy when the mothers could mention the games and describe them on their own. When a mother is able to demonstrate and describe it then it means she has understood and is able to do it with the child.” [1] “What the CHVs would do is to use a group approach to have discussions around these sensitive topics [marital relations] and would not go pinpointing specific mothers.” [1] “The CHVs had a curriculum and it was really their guidance. The manual of session guides was very important because it was a very useful tool for the CHVs to be able to correctly deliver the sessions to the mothers.” [2]	“I had a manual such that when I have gone there it tells me everything. When I feel like am forgetting I refer to the manual. Then things run smooth because human beings can forget.” [B] “When I knew that I will have the sessions on such a day then I could go back to my books—the manuals and then I read about the information for the CHV and also all the activities, and this made me remember what I wanted to say during the session.” [A] “When we started the first session, the mothers gave us their expectations and what they wished for their children to be in future. So we told them how the brains of their children were going to develop and if they developed the brains of their children then they would achieve their wishes.” [A]	“The playing bag that I have in the house will motivate me and the key messages posters that I was given will also motivate me to continue practicing.” [A] “Even a neighbor who was never in the program came to ask what the poster is all about and you explain what it is.” [C] “When he sat and saw other children there, he would have the urge of playing, if it is arranging the shapes and the bottle tops. Even if he didn't arrange them as required, he would want to play. There are days when we were not supposed to meet, he would hold my hands and tell me we go. You know there are a lot of children there, so he feels happy when he sees other children playing.” [A]

*Quotes from 2 FGDs with supervisors are identified as 1 or 2; quotes from 11 CHV interviews and 8 pairs of mothers are identified by sub-county as A, B, C*.

### Question 2

How did Outputs such as delivery fidelity and parental attendance and enactment (i.e., adoption of promoted practices) change during the intervention, and what determined these Outputs?

To answer this research question, we discuss aspects of fidelity, attendance and enactment of practices. Within each topic, we first present quantitative results followed by analyses of their determinants, and under determinants we include relevant quotes from qualitative interviews with parents, CHVs, and Supervisors.

#### Fidelity

**Figure 2:**
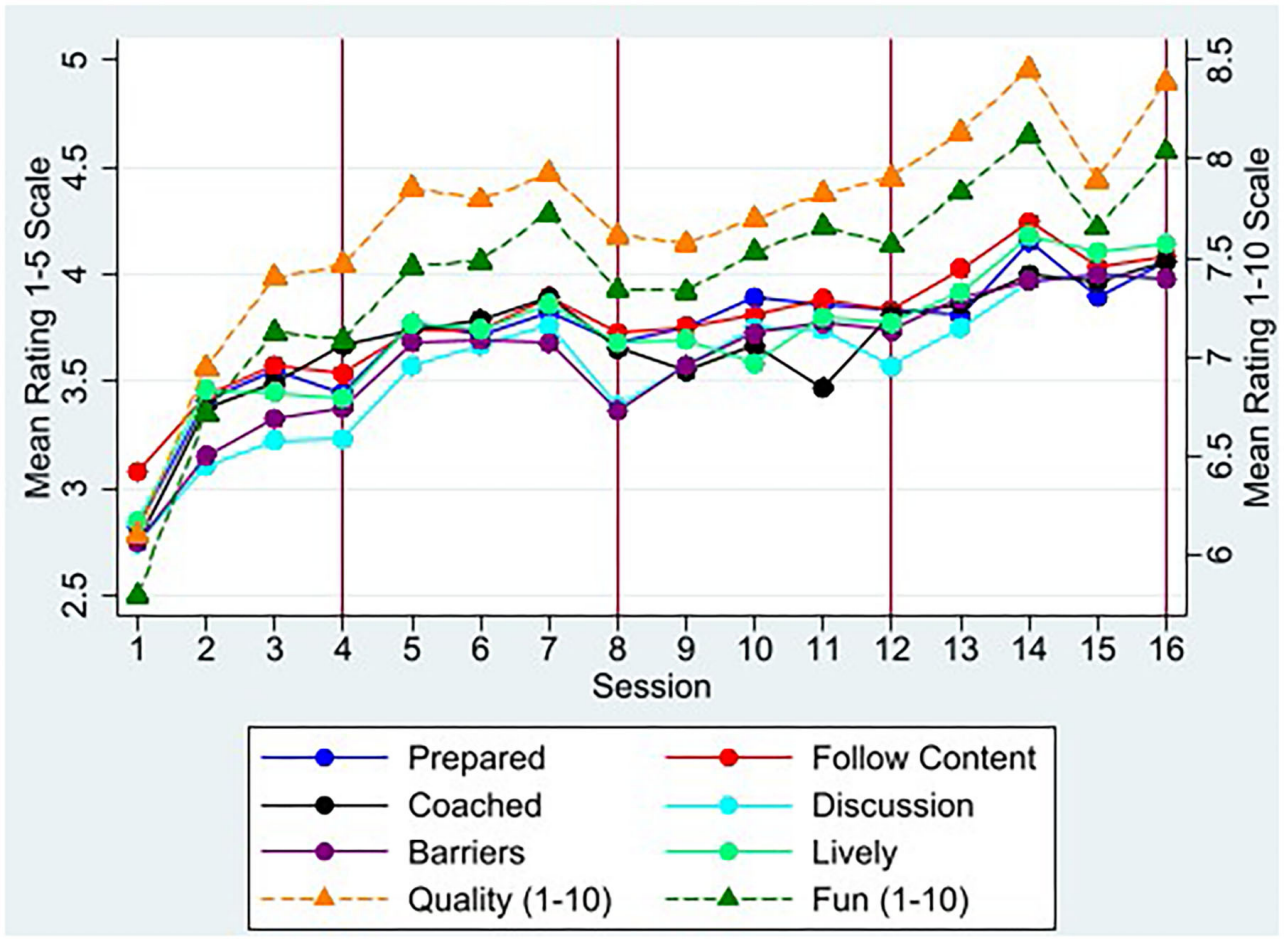
Mean CHV Delivery Scores by Session. Figure plots mean scores from supervisor monitoring forms across all CHVs by session for items measured on a 1–5 scale from “poor” to “excellent” for six of 13 delivery measures using solid lines. *N* = 686 supervisor rating forms across 16 sessions and 40 villages. Some earlier sessions had more than 1 supervisor present. For session “quality” and “fun,” figure plots mean scores on 1–10 scale on right-hand-side axis using dashed lines. All 13 delivery measures show similar upward trends and the full list is in Figure 3. Red vertical lines represent review sessions when sessions were home visits for mixed-delivery arm and group sessions for group-only arm.

Monitoring by supervisors took place as intended during 88% of the 12 (non-review) group sessions across all arms and villages, and 91% of group review sessions were supervised. Fewer home visit review sessions were supervised by necessity (9%). Monitoring forms (*n* = 686) showed gradual and statistically significant improvement across all sessions in all delivery measures (Figure 2). Similar upward trends were seen in self-evaluation ratings made by CHVs (*n* = 2,143; results available upon request). The median session lasted 90 minutes.

**Figure 3:**
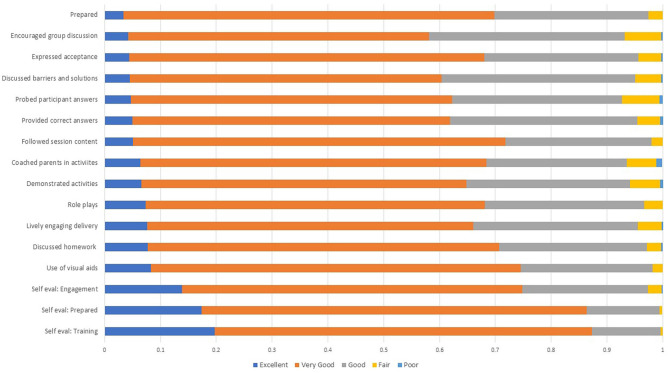
Delivery Ratings: Supervisor and Self-Evaluations. Findings are mean ratings from supervisor or self-assessment monitoring forms across all 16 sessions and 40 intervention villages. *N* = 686 for supervisor monitoring forms; *N* = 2,043 for CHV self-assessment forms that include each home visit.

Most aspects of session delivery were rated good or excellent in 60% of sessions or more by supervisors (Figure 3). The most difficult skills involved facilitating group discussions, and discussing problems and solutions to enacting the new practices. CHVs performed better at following session content, discussing homework, expressing acceptance, being prepared and using visual aids. CHV self-evaluations were in general agreement, if slightly inflated, relative to supervisor ratings for aspects of delivery quality (Figure 3).

##### Determinants of Fidelity

The 40 CHVs from the intervention villages who underwent training and delivered the intervention included 10 males and 30 females, were on average 44 years old (range 26–69), had 11 years education (range 7–14), and 9 years experience as a CHV (range 1–24). All CHV characteristics were balanced across intervention arms. CHVs who scored higher on a knowledge test of ECD after the first training delivered better quality and more engaging sessions, and scored higher in all fidelity outcomes on average (Table 3). CHV age, education, and sex were not consistently related to performance.

**Table 3:** Determinants of delivery fidelity, supervisor, and self evaluations.

	**(1)**	**(2)**	**(3)**	**(4)**	**(5)**
	**Supervisor Monitoring Forms**			**CHV self-evaluation forms**	
**Variables**	**Quality 1–10**	**Fun 1–10**	**Fidelity Index all items**	**Quality 1–10**	**Fun 1–10**
Session (1–16)	0.097^***^	0.099^***^	0.105^***^	0.063^***^	0.070^***^
	(0.011)	(0.012)	(0.012)	(0.013)	(0.011)
CHV is male	0.171	0.179	0.084	0.116	0.173
	(0.125)	(0.126)	(0.119)	(0.236)	(0.194)
CHV above median: education	0.208	0.217^*^	0.148	−0.029	−0.128
	(0.130)	(0.123)	(0.132)	(0.235)	(0.173)
CHV above median: age	−0.031	−0.071	−0.095	−0.031	0.350^**^
	(0.126)	(0.120)	(0.124)	(0.235)	(0.153)
CHV above median: baseline knowledge	0.380^***^	0.317^***^	0.218^*^	0.447^**^	0.229
	(0.123)	(0.112)	(0.124)	(0.201)	(0.156)
Constant	6.959^***^	6.414^***^	−1.040^***^	6.982^***^	6.979^***^
	(0.214)	(0.199)	(0.188)	(0.386)	(0.208)
Observations	684	684	686	2,043	2,043
R-squared	0.332	0.278	0.367	0.056	0.144
Number clusters	40	40	40	40	40

*Results from monitoring forms filled out by CHVs or supervisors following each session in the 40 intervention villages. Robust standard errors in parentheses clustered at village level*.

*** *p < 0.01*,

** *p < 0.05*,

* *p < 0.10. Columns 1–3 present OLS regression results from supervisor monitoring forms for the delivery outcomes specified in each column. Columns 4 and 5 present OLS regression results from self-evaluation forms filled out by CHVs for delivery outcomes. Columns 1, 2, 4, and 5 are measured on a scale from 0 to 10. Column 3 presents results from a composite index estimated with factor analysis that includes 13 fidelity items assessed in the supervisor monitoring forms with a 1–5 scale. Those 13 items are also listed in Figure 3*.

During post-intervention interviews, CHVs and supervisors were asked to comment on training, supervision and monitoring of performance. Their answers reveal important determinants of fidelity. Illustrative quotes are summarized in Supplementary Table 1. CHVs responses showed a need for more practice demonstrating responsive play and communication, and facilitating a group discussion on problems enacting the messages. They were more familiar with the key messages on nutrition and hygiene. CHVs became more proficient and confident in their session delivery after the midline training when SWAP staff trained them in smaller groups and in the language of delivery, and with refresher meetings in the sub-counties twice a month (Supplementary Table 1). Their post-training knowledge scores from the first to the midline training increased from a mean of 57.2% to 60.8% correct, though this was not statistically significant (*p* = 0.17). Their performance scores in delivering a mock session after training served as a way to identify 30% of CHVs who needed extra training.

All 11 interviewed CHVs mentioned ways the supervisor feedback and opportunity to evaluate themselves after sessions was helpful, and reported having prepared themselves ahead of sessions. CHV descriptions of how they demonstrated responsive talk were thorough: 8 out of 11 CHVs described at least 3 key components such as letting the child show what he/she is interested in, and then the parent repeats or expands verbally. However, descriptions of how they demonstrated responsive play were incomplete and none was able to name three key components of responsive play; they often correctly mentioned it was letting the child choose playthings, but then incorrectly required the child to follow the parent's lead. CHVs said they initially found it difficult to facilitate group discussions, but later developed a style to encourage shy mothers and sum up the best solutions at the end. Interviews with supervisors showed they recognized early on that CHVs would benefit from periodic refresher trainings ahead of sessions (Supplementary Table 1).

#### Maternal Attendance

**Figure 4:**
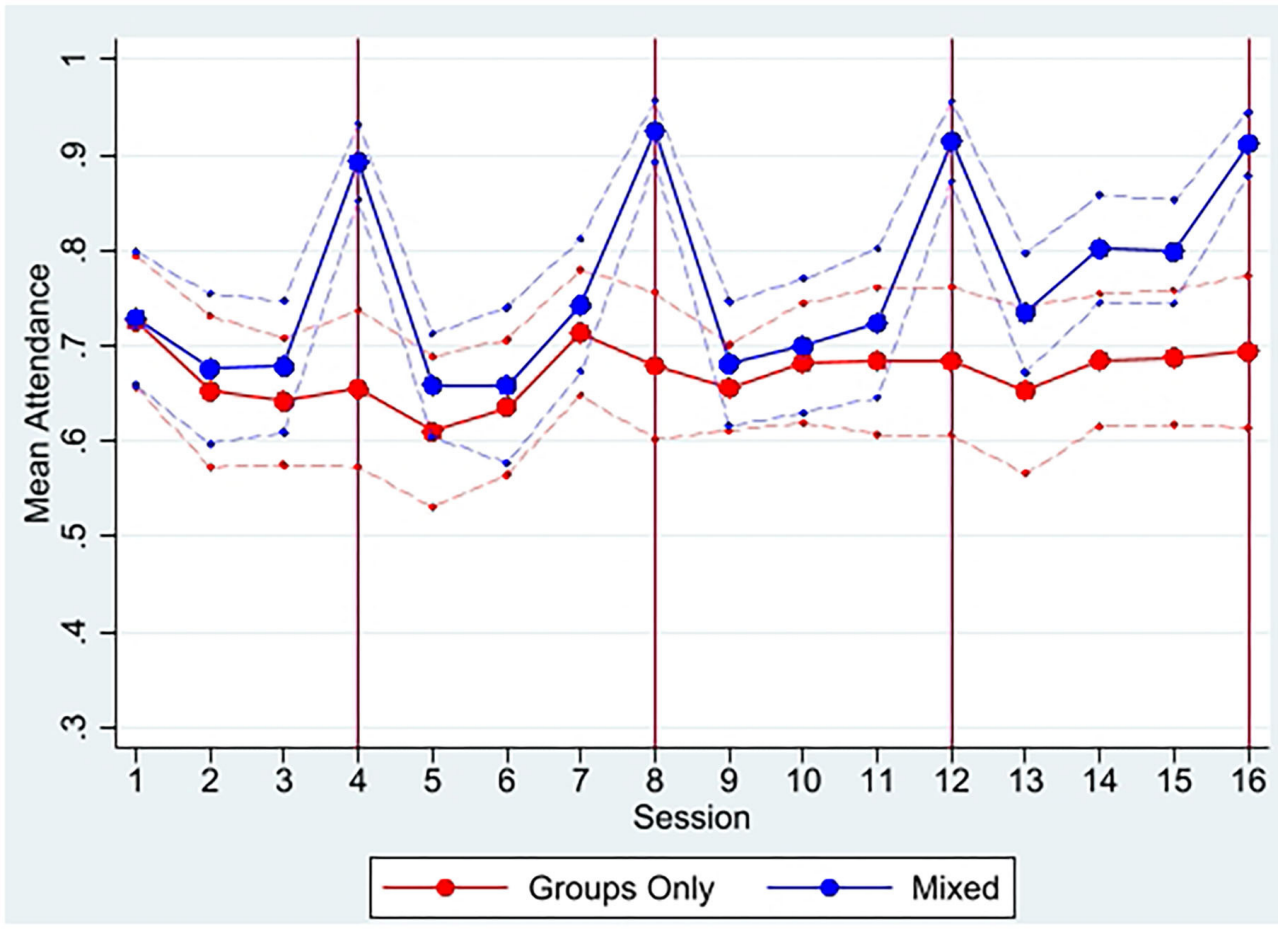
Mean maternal attendance by study arm and session. From attendance monitoring data. Dashed faded lines represent 95% confidence intervals; red vertical lines represent review sessions when sessions were home visits for mixed-delivery arm and group sessions for group-only arm. The only sessions with statistically significant differences in attendance at 95% level or higher are the four review sessions (4, 8, 12, and 16).

Among the 776 intervention households, 97% of mothers attended at least one Msingi Bora session, with no differences by intervention arm (*p* = 0.142). The median mother attended 13 of 16 sessions (IQR 8–15 sessions) with a median group size of 13 mothers at a group meeting (IQR 10–15 mothers). The mixed-delivery model had higher overall attendance, with an average of 74 percent of mothers attending a given session, compared to 64 percent of mothers in group-only villages. The bulk of this difference comes from the review sessions, which were home visits for the mixed-delivery arm (and thus, mothers did not have to travel to these sessions). These sessions were the only ones in which attendance was statistically different across arms (Figure 4).

##### Determinants of Maternal Attendance

Maternal attendance to the group sessions was higher in households with a father present, and with higher baseline FCI stimulation scores (Table 4). More educated mothers and those with a greater distance to walk had lower attendance. Mothers also attended more sessions in villages where CHVs were rated higher on fidelity measures by supervisors (Table 4).

**Table 4:** Determinants of maternal attendance to common group sessions.

	**Mother Attendance**
	**Mean/SE**
**Experimental treatments**	
Mixed-delivery model	0.335 (0.362)
**CHV/delivery characteristics**	
CHV is male	0.490 (0.420)
CHV above median: education	−0.275 (0.371)
CHV above median: age	−0.396 (0.349)
CHV above median: baseline knowledge	0.126 (0.356)
CHV Fidelity Factor Scores (mean = 0, SD = 1)	0.795 (0.302)^**^
**Household characteristics**	
Baseline child ability factor	0.210 (0.130)
Baseline FCI score	0.354 (0.169)^**^
Female child	0.128 (0.266)
Birth order	0.000 (0.096)
Mother's education (years)	−0.230 (0.054)^***^
Father in household	0.563 (0.296)^*^
Wealth asset index (mean = 0, SD = 1)	0.102 (0.076)
Distance to venue in Km	−0.691 (0.306)^**^
Constant	10.390 (1.289)^***^
Observations	*N =* 708
R-squared	0.132
Mean dependent var	8.341
Number clusters	40

*Robust standard errors in parentheses clustered at village level*.

*** *p < 0.01*,

** *p < 0.05*,

* *p < 0.1. Results are from multivariate OLS estimation of the number of group sessions attended out of a maximum of 12*.

During post-intervention interviews, both Mothers and CHVs were asked what encouraged mothers to attend. The frequencies of commonly mentioned reasons and representative quotes are seen in Table 5. Benefits to the child were recognized by both groups of participants, but CHVs frequently mentioned incentives and their reminders to the mothers, whereas mothers frequently said that socializing with others drew them to sessions. An equal number mentioned the content of the program. No mother mentioned reminders or incentives as spurring their attendance.

**Table 5:** Barriers and enablers for caregiver attendance—Mother and CHV perspectives.

	**Mothers (*n =* 8 pairs)**		**CHVs (*n =* 11) comments on mothers**	
	**Frequency out of 8**	**Quotes**	**Frequency out of 11**	**Quotes**
**What made it easy to attend?** - Benefits to Child - Socializing - Program Content - Reminded by CHV - Incentive	8 5 5 0 0	“I wanted my child to become a clever person… that's what really motivated me.” [A] “We were taught how to talk to our husbands in a polite way even if we were wronged. I wanted to have a peaceful family where there is respect and love.” [A] “I came to realize that our children had started making friendship among them. You find that when they are playing one child would take his/her play item and give to the other child.” [B] “I met other fellow women when we went for group sessions as compared to home visits.” [C]	8 0 5 7 8	“When they see the progress their children are making they decide to come to know what is next.” [B] “They felt it was really helping them by teaching them a lot about ways of living and of loving.” [C] “I would arrive early so that I can call them again. I would even walk to participants who were my neighbors and remind them to leave for the meeting.” [B]
**What made it difficult to attend?** - Travel/time - Competing demands, e.g., chores - Other, e.g., want incentives	6 2	“The meeting place was far away and we had to walk for long, so sometimes it was very difficult.” [B]	0 1 1	“People sometimes suffer hunger, I used to make them a cup of tea and one doughnut. The mothers were also busy.” [C] “Some refused completely, they would say if there is nothing that I am going to be given then I will not come back.” [A]

*Frequency refers to the number out of 8 pairs of mothers and out of 11 CHVs who offered an answer. Quotes from 8 pairs of mothers and 11 CHV interviews are identified by sub-county as A, B, C*.

#### Parental Enactment

Mothers and CHVs were asked about barriers and enablers to enacting the new program practices (see Supplementary Table 2). Both mothers and CHVs reported it was sometimes difficult for mothers to find new play things and to find 15 min daily to play with their children, but learning games with sticks and learning how to talk about pictures in a book facilitated responsive play and talk practices.

Many of the responses to enactment of specific practices by mothers, especially about play and communication (Supplementary Table 2), triangulated well with answers by CHVs and with relevant HOME Inventory items (Table 6). For example, 8 out of 8 mother pairs said they had a playbag, and 7 out of 8 said they added new playthings. These responses were largely corroborated by CHVs, and by the full sample's responses to related items on the HOME Inventory at endline (Table 6). Similarly, 6 out of 8 interviewed mother pairs said they look at a book with their child daily; on the HOME, 82% of intervention mothers vs. 23% in the control group reported looking at a picture book with their child in the past week.

**Table 6:** Maternal enactment of new practices.

	**Intervention Mean**	**Control Mean**	**Diff Intervention vs. Control**
**Responsive Play (HOME)**			
There is a bag/box where the child keeps their play things	64.9%	21.6%	0.431^***^
Gross motor objects available. e.g., ball, rope, ring, flat stone	46.7%	29.0%	0.175^***^
Push or pull toys. e.g., pull with string, push box	26.7%	15.9%	0.114^***^
Dramatic play materials, e.g., doll, transport, household items	75.3%	55.4%	0.204^***^
Simple eye-hand coordination materials (e.g., rattle)	47.2%	32.4%	0.153^***^
Complex eye-hand coordination materials (e.g., sticks and caps)	38.8%	15.1%	0.246^***^
Did you find/make something new for your child to play with?	33.2%	12.8%	0.202^***^
Child played with slightly difficult materials in the last week	61.1%	44.6%	0.170^***^
**Responsive Communication (HOME)**			
The child has looked at a picture book in the last week	82.2%	23.3%	0.595^***^
The child has three or more picture books	8.2%	2.3%	0.062^***^
The mother talks with the child when she is busy at home	73.6%	70.5%	0.044
The mother talked about pictures in a book, calendar, etc.	76.0%	38.1%	0.386^***^
Did you tell your child a story?	36.4%	18.2%	0.185^***^
**Food (Food categories eaten by the child last 24 h)**	4.279	4.057	0.222^**^
**Observations**	719	351	

*Each row reports the means for the intervention arms combined vs. the control arm for different HOME items related to responsive play and communication. The last column reports the adjusted difference between the intervention arms and the control arm for each item, adjusted differences not always identical to difference in unadjusted means presented in table. Adjustments include child age, sex, and birth order, household wealth and maternal education. Standard errors are clustered at the village level. Significance levels:*

*** *p < 0.01*,

** p < 0.05, ^*^p < 0.10. ^*^Statistical significance levels as clarified in notes to table.

### Question 3

What features of inputs and outputs were in turn associated with child and parent outcomes, and how did this differ by delivery mode?

After combining endline survey and program implementation data, we found that aspects of both the delivery agents and their performance were often related to parent and child outcomes (Table 7). For example, CHV age and initial-training knowledge of ECD were positively related to better HOME scores as well as to child developmental outcomes, but not to dietary diversity. CHVs with higher performance ratings by supervisors were associated with better HOME scores, but we did not see a similar relation with any child outcomes.

**Table 7:** Determinants of final outcomes.

	**(1)**	**(2)**	**(3)**
	**HOME scores**	**Dietary diversity**	**Child dev. index**
**Experimental treatments**			
Mixed-delivery model	0.045	−0.200^**^	−0.098
	(0.131)	(0.086)	(0.148)
**CHV/delivery characteristics**			
CHV is male (=1)	−0.122	0.106	0.053
	(0.137)	(0.089)	(0.178)
CHV above median: education	−0.086	−0.067	0.020
	(0.095)	(0.106)	(0.175)
CHV above median: age	0.373^***^	0.024	0.480^***^
	(0.096)	(0.095)	(0.176)
CHV above median: baseline knowledge	0.212^*^	0.066	0.515^***^
	(0.111)	(0.090)	(0.186)
Fidelity factor index scores	0.245^***^	0.029	0.036
	(0.081)	(0.085)	(0.159)
**Household characteristics**			
Baseline child ability factor	0.043	0.010	0.192^***^
	(0.039)	(0.027)	(0.061)
Baseline FCI score	0.119^**^	0.020	−0.006
	(0.046)	(0.042)	(0.043)
Female child	0.091	0.008	0.116
	(0.083)	(0.066)	(0.079)
Birthorder	0.065^***^	0.017	−0.007
	(0.024)	(0.026)	(0.026)
Mother's education (years)	0.053^***^	0.041^**^	0.047^**^
	(0.015)	(0.016)	(0.020)
Father in household	0.075	−0.014	0.013
	(0.096)	(0.059)	(0.083)
Wealth asset index (mea*n =* 0, SD = 1)	0.055^*^	0.043^**^	0.048
	(0.030)	(0.019)	(0.029)
Distance to venue in Km	0.044	−0.011	0.065
	(0.132)	(0.086)	(0.147)
Constant	−0.168	−0.102	−1.071^***^
	(0.274)	K (0.232)	(0.305)
Observations	710	709	710
R-squared	0.132	0.123	0.182
Number clusters	40	40	40

*Robust standard errors in parentheses clustered at village level*.

*** *p < 0.01*,

** *p < 0.05*,

* *p < 0.10. Results are from multivariate OLS regressions of final project outcomes as a function of household and CHV characteristics. All models control for strata (sub-county) fixed effects. Column 3 is an index constructed with factor analysis of four child development outcomes at endline including cognitive, receptive language, expressive language, and socioemotional outcomes. All outcomes are standardized to be mean 0 with SD 1*.

While both intervention arms saw improvements in key outcomes relative to the control arm, the group-only arm generally outperformed the mixed-delivery arm in child impacts, though differences were rarely statistically significant (14). The only practical difference across intervention arms were the four review sessions, which were delivered in groups in the group-only arm and through home visits in the mixed-delivery arm. Interviews with CHVs, supervisors and mothers asked their perspectives on group meetings and home visits (Table 8). CHVs and supervisors had positive things to say about both meeting formats, but appeared to have a preference for home visits, where they were able to maximize attendance, provide greater direct supervision of the mother's practices, and appreciated that “you will know the truth, whether she is doing it or not.” Conversely, mothers clearly preferred the group meetings, where they felt their children were able to socialize more and learn how to play with others, and they felt better supported by other mothers. Mothers viewed home visits somewhat as an inspection and a test of their uptake, using phrases such as “The CHV would come inspect the play bag,” and “They would ask me questions from the poster and I would answer.”

**Table 8:** Mother, CHV, and supervisor perceptions about home visit vs. group meetings.

**Interviewee**	**Quotes**
Mothers	Home Visits: “CHV would come inspect the play bag, pour the contents down and what she sees needs improvement then she would tell me. And she would also ask me to recall the things that we were trained just so she would know if I can remember.” [A] “When they came they asked for the five key messages poster. Then they would ask me questions from the poster and I answer. After that we take the play bag and we spread a mat down and start playing with the child.” [C]
	Group Meetings: “I came to realize that our children had started making friends among them. This made me think that we have been confining our children in the house for a long time, but they need to play and share play items among the other children from other areas.” [B] “Before the program started, my child did not like people and was never friendly. Someone could not even touch her. Since we started coming for the session, I saw that the child was very jovial and she started being social to people and even talk to strangers.” [B] “Sometimes there is something that I did not understand in a session and I could ask my buddy to explain it to me. Our children had gotten used to one another so when I am busy at home or I want to go somewhere, I can take my child to play with my neighbor's child.” [B] “I preferred the group meetings because my child is shy when he sees a new face but when he is among the other children he will just be okay and active. I also met other fellow women when we went for group sessions.” [C] “The group is good because you can take the child and he would play with others. People would exchange ideas and someone might mention something you did not know.” [A] “Children enjoyed playing together and so even us parents were happy when we saw children enjoying playing.” [A]
CHVs	Home Visits: “Home visit was good because we would get the father; and also the children's grandmothers.” [A] “Not all participants will come for the group sessions. When you go to their houses, you will find them.” [B] “In home visits you will know if the parent has a play bag for the child, if she has placed it where it is supposed to be, and if she spends time to play with the child during the day. How is the hygiene? How's the play and even food? You can ask about the progress; you will know the truth, whether she is doing it or not.” [B] “It was very tiresome to go for home visits. I walked a lot because I had to visit 21 households.” [B] “Home visits you can't do in 1 day; it takes a long time. Group sessions can be done in a day.” [C]
	Group Meetings: “They could open the discussion and get answers by themselves. Someone can say ‘I cannot manage to do this because of poverty.’ The CHV could leave them to discuss and then just conclude at the end. One mother could just tell her ‘just try and buy some seeds and then plant vegetables. So you will eat some and sell some vegetables and get money to buy what you need’.” [C] “Once people share you can see some of them nodding their heads and saying that they have also gone through that. And that is when you ask them to share what they went through and they stand up and share it out. And we assure them that what they have shared is confidential.” [C] “Group session is easy because when someone does not know something that she can be reminded by someone in the group.” [A] “Group sessions were easy because the weaker women will learn from the ones who are doing the right things.” [A] “Another advantage is that mothers who are in the session will ask you questions and you will answer to many people at once.” [A] “There are also some children who are very keen and when they see what the other child does then they will also do the same.” [A] “In group sessions, you will just put everyone together and finish at once.” [B]
Supervisors	Home Visits: “Home sessions were nice because you find a mother at home and she is ready to talk. We see how she is doing, we find she has put the poster on the wall.” [1] “The home visits are necessary because you can find the mother and the child waiting for the session.” [1] “Home visit are very important because of the support that they get from the other relatives.” [1] “Sometimes in the group meetings mothers would not talk but when you go to their homes now they would talk.” [1] “Home visits were good. Doing home visits you will capitalize on time when he/she is at home.” [1] “After the session we talk about it [the challenge identified] and it is between the three of us and no one will feel intimidated.” [1] “In home visits you will be able to find some mothers have not hanged the poster on the wall and you will find out she is not doing what you taught her in the session. It is easy because you get to see what the mother is doing at home.” [1] “I could see some tension in a group discussion. At home they were more relaxed, seated, and chatting.” [1] “The home session helped participants who were not talkative in the groups. Now because it is between me and you, it will force you to talk.” [1] “At home we could handle some issues that we could not handle in a group. Some felt if they shared something people might talk about it.” [1] “During the home visit they would really be committed. The advantage is you are able to understand the mother's challenges.” [2] “You are engaging with the person personally so that information can sink deeply.” [2] “There is also a lot of interruption during the home visit since children would be coming home for lunch, or visitors would come.” [2]
	Group Meetings: “Group visits are easier because you are looking at different aspects of the manual. At home you only see she is not doing well.” [1] “In groups, we find that there are those active mothers that would feel free to share their experiences and there are those shy ones also. Now what the CHVs would do is to use a group approach around these sensitive topics and would not go pinpointing mothers.” [2] “There was a lot of social support within the groups to be able to do these things well.” [2] “The advantage is they can learn from others.” [2] “The group sessions are easier to supervise.” [2] “In a group, when you have 20 people, you take only 1 h, not 20 has in home visits.” [2] “CHVs were happy they were meeting the mothers in a central place; they are used to visiting mothers in their households.” [2]

*Quotes from mothers and CHVs are identified by letter according to their sub-county; Supervisors are identified by the number of their FGD. All quotes that participants expressed were included*.

## Discussion

This implementation evaluation of our cluster randomized effectiveness trial testing an ECD parenting program in rural Kenya demonstrated acceptability of the program content and format, high maternal attendance to group sessions, improved fidelity over time, and links with final outcomes. These findings demonstrate the importance of implementation quality and delivery performance. We did not present results on the inclusion of fathers, who attended sessions at low rates, and their inclusion had no measurable impacts. Further details on including fathers will be discussed in another paper.

Regarding the first research question, the curriculum, which was focused on mothers' interaction with their children to provide responsive play and communication, addressed the needs of both parents and CHVs who knew little about the role of stimulation in mental development starting at birth (18, 19). This was confirmed by low baseline scores on the FCI, as well as by post-intervention qualitative interviews with parents, CHVs and program supervisors, which revealed that mothers were not accustomed to playing with or providing playthings for their children, and that CHVs were not used to demonstrating games for children. CHVs found acceptable the Manual that laid out a structured set of activities to be conducted each session. CHVs and caregivers liked the practical approach to demonstrating and coaching new play and communication interactions.

Other programs use a different curriculum, focusing on teaching specific skills to the child to be repeated by the mother and child as homework (30, 31), or counseling individual mothers after identifying gaps in their responsive stimulating interactions with children (23). Teaching specific age-appropriate skills would not be suitable for a large group of mothers with children of different ages. Likewise, counseling does not always suit group sessions. Instead, coaching after a CHV demonstration or having some mothers demonstrate while others watch was appropriate for our setting. Other program developers and implementers have also adapted the content and delivery of well-known parenting programs such as Reach Up (32) and Care for Child Development (CCD) (33) to deliver them in groups in rural areas with low-skilled parents and providers, including making the program more structured (32). We therefore attribute acceptance of the content and delivery format of the Msingi Bora program to these aspects of the curriculum and its delivery.

Regarding implementation fidelity, CHVs' experience delivering sessions and frequent supervisory feedback over the 8-month intervention led to significant improvements in CHV delivery performance and enjoyment of mothers and CHVs with the program. A key finding from our implementation evaluation is that CHVs who grasped the content more thoroughly from the first training tended to score better on measures of delivery performance, and were associated with significantly improved parenting practices and child development at endline. Similarly, CHVs who delivered better sessions had greater attendance, and their villages had higher HOME scores at endline, though not better child developmental outcomes. These results are highly encouraging given that our study trained low-skilled delivery agents and used the existing local infrastructure in a disadvantaged setting. It is also encouraging that both male and female CHVs were able to deliver these sessions, and CHV sex was not a consistent determinant of program quality or final outcomes.

As mentioned above, the quality of CHV session delivery increased as the program unfolded. This is potentially a result of a course-correction in our training program with the introduction of monthly refresher trainings beginning with session 4. The initial plan for a baseline and midline training to cover half of the 16 total sessions apiece proved to be insufficient for our delivery agents. Because CHVs were unfamiliar with the practice of responsive play and communication, they initially found it difficult to demonstrate and coach these actions. Extensive practice during training and the introduction of monthly refresher trainings was required to overcome the habit of instructing and directing children. Likewise, CHVs were unfamiliar with facilitating open discussions with groups of parents, probing them for more descriptions of their experiences and encouraging them to solve problems together. What helped was to have them watch each other, especially those more skilled, practice facilitating small group discussions. Interviews with parents, CHVs and supervisors recognized the improvement in quality that developed over time. Other implementers have similarly highlighted the importance of provider competence and the need for more training and supervision than initially expected (11, 19, 34–36).

Parental attendance to the group sessions was an anticipated challenge in our dispersed and disadvantaged rural setting. Indeed, mothers with more education and who lived farther from a meeting place had lower attendance on average, suggesting an opportunity cost of attendance. However, the effect of distance was small: each additional kilometer to travel resulted in −0.7 fewer group sessions attended, and the median distance mothers had to travel was 0.65 kilometers. Although maintaining high attendance is an issue with many group programs (9), perceived benefits became a motivator over time. Until mothers saw the benefits of the program, reminders and incentives were likely required, but their importance was not recognized by mothers by the end of our program.

Mothers' attendance and receptiveness to the program content translated into high reported uptake of the recommended behavioral practices as measured by the HOME Inventory at the endline survey. Interviews with a subsample of mothers indicated that most had a playbag for their child's playthings, and spent time playing with them, though initially they found these practices difficult (e.g., due to competing priorities such as household chores). Less frequently than requested, they added new materials to the bag. After learning how to engage in responsive two-way talk with their child, they found this relatively easy. When given a picture book in a later session, they were able to apply the responsive talking method to it rather than simply reading. Quotes from mothers and CHVs (Supplementary Table 2) confirm the gradual uptake of these important practices, especially as mothers noticed positive benefits for their children.

Perhaps the most surprising result of our study is the relative advantages shown by the group meetings to improve children's final outcomes. The interviews with mothers clearly highlight group camaraderie and opportunities for peer learning among the children as relative benefits of the group format, and caution against a model that utilizes home visits if they engender a feeling of mothers being under inspection.

A key limitation of our study is that, though interviews point to convincing reasons, we cannot say with certainty why children in the group arm outperformed the mixed-delivery arm. Another study limitation is that we have only coarse measures of CHV characteristics and expertise, which constrains our ability to predict who might make the best delivery agents.

Our results show that a responsive stimulation and nutrition education intervention featuring large group sessions delivered by para-professional community health workers can benefit multiple child and parent outcomes. An upfront investment in training local trainers and delivery agents, and regular supervision of delivery of a manualized program, appear key to our documented success. Our results represent a promising avenue for scaling similar interventions in low-resource rural settings to serve families in need of ECD programming.

## Data Availability Statement

The datasets presented in this article are not readily available because the de-identified datasets generated during the study along with statistical plan and analytic code will be available from the corresponding author on reasonable request at the end of the 5-year project after all planned articles have been accepted for publication. We will make the data without identifiers available to users only under a data-sharing agreement that provides for: (1) a commitment to using the data only for research purposes and not to make the attempt of identifying any individual participant; (2) a commitment to securing the data in case there are still some sensitive variables after the identifiers have been removed, by using appropriate computer technology; and (3) a commitment to destroying or returning the data after analyses are complete; and (4) a commitment to not publish any information that is not treated at the aggregate level so that no specific characteristics can be linked to small communities. Requests to access the datasets should be directed to jluoto@rand.org.

## Ethics Statement

The studies involving human participants were reviewed and approved by Ethics approval was obtained from Maseno University in Kisumu, Kenya, and RAND. Written informed consent to participate in this study was provided by the participants' mothers and fathers, or legal guardian/next of kin.

## Author Contributions

JL and IL secured the funding, designed the study, and performed the quantitative analyses. JL, IL, and FA wrote the first draft of the manuscript. FA, DS, JL, and IL trained SWAP's trainers into the intervention content. RO and EA became lead trainers for CHVs and managed the study's implementation throughout. FA and JL trained the qualitative researchers. FA, DS, and RZ analyzed the qualitative data. FA and DS reviewed and provided critical input to study design and conceptualization. All authors reviewed and contributed to writing the final draft.

## Conflict of Interest

The authors declare that the research was conducted in the absence of any commercial or financial relationships that could be construed as a potential conflict of interest.

## Supplementary Material

Additional Files Supplementary Material file includes CARE Guidelines to show where in manuscript each CARE item is described, as well as Supplementary Tables 1, 2 that are too large for inclusion here.

## Abbreviations

CARE: Consolidated Advice for Reporting ECD implementation research
CHV: Community Health Volunteer
ECD: Early Child Development
FCI: Family Care Indicators
HOME: Home Observation for Measurement of the Environment
LMIC: Low and Middle Income Country
MOH: Ministry of Health
NGO: Non-Governmental Organization
NCF: Nurturing Care Framework
SWAP: Safe Water and AIDS Project.
