# Supplementary material for: An Implementation Evaluation of A Group-Based Parenting Intervention to Promote Early Childhood Development in Rural Kenya
Source: Front Public Health. 2021 May 5;9:653106. doi: 10.3389/fpubh.2021.653106 (PMC8131637; doi:10.3389/fpubh.2021.653106)
Supplement: Additional Files — Supplementary Material file includes CARE Guidelines to show where in manuscript each CARE item is described, as well as Supplementary Tables 1, 2 that are too large for inclusion here. [file Table_1.docx]

**CARE Reporting Guidelines. Consolidated Advice for Reporting ECD Implementation Research**

| **Section of the Report** | **Item Number** | **Item Label** | **Description** | **Location in Manuscript** |
| --- | --- | --- | --- | --- |
| Introduction | 1 | Previous evidence about the intervention | (a) Briefly describe the nature and severity of the problem(s) being addressed, explaining why the intervention is needed. (b) Briefly describe what is already known about the effectiveness of similar interventions previously implemented or interventions that target similar outcomes. | Background page 4 |
|  | 2 | Effectiveness of the present intervention (if known) | If the effectiveness of the specific intervention has been evaluated, please include: (a) Summary of the research design (e.g., randomized controlled design, quasi-experimental design). (b) Summary of the findings pertaining to the primary and secondary outcomes (e.g. child outcomes, caregiver behavior changes, caregiver outcomes). | Background page 5 |
|  | 3 | Rationale for the implementation research | Explain why the implementation research is needed for the specific intervention. | Background page 4 |
|  | 4 | Aims and objectives of the implementation research | List the aims and objectives of the implementation research. | Background page 5 & research questions page 6 |
| Methods | 5 | Context of implementation for the intervention | Briefly describe the setting for the implementation research and state the following:  (a) The location and the social, economic and policy context. (b) Dates for when the implementation research was conducted. (c) Information about ethical approvals and consenting procedures. | Methods: Setting & participants; ethical approval pages 6-7 |
|  | 6 | Implementation strategy for the intervention | Describe the implementation strategy for the intervention. A ‘Logic Model’ organizing the inputs and expected outputs is recommended. | Figure 1 |
|  | 7 | Implementation recipients for the intervention | Include a summary about the recipients of the intervention. | Methods: Setting & participants, pages 6-7 |
|  | 8 | Intended intervention content | Describe the intervention in the intervention condition and the comparison condition. Include:  (a) Summary of any formative research or piloting to design or adapt the intervention. b. Information about the curriculum used (i.e., adaptations, translations, manuals, job aides) and content.  (b) Describe the theory of change, conceptual model, or framework on which the intervention is based (e.g. behaviour change theory).  (c) List any behavior change techniques employed (e.g., information sharing, problem solving, performance, social support, distribution of materials, visual aides). | Methods: Implementation strategy. Content and delivery strategy. Pages 7-9 |
|  | 9 | Changes to intervention content | Provide information about, and explain, the reasons for changes made to the intervention in the intervention condition and in the comparison condition after the intervention was initiated. | NA |
|  | 10 | Intended intensity/total exposure to the intervention | Provide information for the intervention condition and the comparison condition) broken down by:  (a) Length of contact sessions or length of day (e.g., in a group care intervention or in a home visitation intervention). (b) Number of contact sessions. (c) Duration of contact over time. (d) Frequency of contacts (e.g., daily, weekly, fortnightly, monthly). | Methods: Implementation strategy. Content and delivery strategy. Pages 7-9. |
|  | 11 | Personnel involved in supporting the implementation of the intervention (e.g., coordinators, trainers, supervisors, and ancillary staff) | Briefly describe information about personnel who may have been involved in supporting the implementation of the intervention This may include implementing organizations and/or government structures.  For example in the health sector personnel may include managers of health facilities or trainers of community health worker, in the education sector personnel may include head teachers or monitors of schools. | Methods: Setting and participants- Supervisors. Pages 7 and 10. |
|  | 12 | Personnel delivering the intervention (e.g. mother leaders, community health workers, teachers) | (a) Describe the delivery agent for the intervention (e.g., who they are, background, credentials, recruitment process, roles and responsibilities, time spent in service, and any previous training received).  (b) Describe how the delivery agents were trained and supervised to deliver the intervention. Include any information about standardization of training (e.g., training manual), who were the trainers, duration, training techniques. (c) Describe how skills of the delivery agent were maintained over time (e.g., coaching, refreshers, by whom). (d). Describe how the acquisition of skills of the delivery agents post-training was measured. (e) Provide information about the workload of the integrated intervention in the existing workload (e.g., hours of work, remuneration, incentives, compensation, and client ratio). | Methods: Setting and participants- Delivery agents; Supervisors.  Delivery personnel; supervisory personnel. Pages 6-7 and 9-10. |
|  | 13 | Methods to assess fidelity regarding delivery of intervention | Describe the methods and tools used to assess fidelity of intervention delivery and receipt of intervention by recipients (e.g., self-report, observation, competency tests, checklists, and monitoring records on recipient participation), sources of data, and at which stage of the project implementation was fidelity assessed. If relevant, describe whether these data were collected in the comparison conditions. | Methods: Fidelity page 11. |
|  | 14 | Methods to assess understanding and enactment of intervention skills by recipients | Describe the methods and tools used to assess the understanding of intervention content (e.g., knowledge about child development milestones) and the enactment (include any intended or planned actions) of intervention skills by the recipients (e.g., qualitative focus group discussions or in-depth interviews, observations of the care and learning environment of the child, caregiver-child interactions) during the course of the intervention. If relevant, describe whether these data were collected in the comparison conditions. | Methods: Caregiver attendance, acceptance, and enactment of practices, page 11. |
|  | 15 | Implementation research data collection team | Describe the data collection team with respect to credentials (specify if the team was internal or external to the intervention), training, quality assurance (e.g., reliability). | Methods: Data collection and analysis plan,  Table 1. Page 11-12. |
|  | 16 | Sampling and data management procedures | (a) Describe the sample size, sampling strategy, recruitment, timing, and incentives.  (b) Data management and cleaning, and for any qualitative data include information on translations and transcriptions. | Methods: pages 9-13. |
|  | 17 | Plan of analysis for implementation data | Describe the plan of analysis.  For quantitative methods this may include a description of the statistical tests employed to compare outcomes between groups, any adjustments, sub-group analyses, and methods to handle missing data. For qualitative methods this may include a description of the process to derive themes/codes and sub-themes/codes, charting or mapping of linkages between themes, and triangulation of themes/codes. | Methods: Data collection and analysis plan,  Table 1. Pages 11-13, 24. |
| Results | 18 | Results of the implementation evaluation | Present the results for all variables and where needed include figures, tables, and quotes for qualitative findings. | Results.  Tables 2 – 9  Figures 2 - 4 |
| Discussion | 19 | Interpretation of findings of the implementation evaluation | Provide interpretation of the findings of the implementation evaluation: (a) Describe influencing factors in the implementation context that may have influenced the implementation and how these were utilized/ addressed during the intervention implementation. Consider any benefits, harms, unexpected results, unintended consequences, barriers and enablers, problems, and failures. Include a description about the generalizability of the intervention implementation in different settings.  (b) How do these data compare with similar interventions or interventions intended to target similar outcomes, and how can these data inform future intervention work? | Discussion, pages 18-20. |
|  | 20 | Strengths and limitations of the implementation research | Describe the strengths and limitations of the implementation evaluation. | Discussion page 20, Conclusions page 20. |
|  | 21 | Scalability and sustainability of the intervention and implementation strategy | Consider any implications of the findings for implementing at a larger scale (e.g., delivery agents, training, monitoring) and sustainability. | Discussion and conclusion, pages 19-20. |

### Supplementary Table 1: Illustrative CHV and Trainer/Supervisor responses to semi-structured interviews concerning training, supervision and delivery

|  | CHV (n=11 interviews) | | Trainer/Supervisor (2 FGDs) |
| --- | --- | --- | --- |
| Item | Freq (out of 11) | Quotes | Quotes |
| ***What was good and poor about your training?*** | | | |
| Helpful/Good elements:  Manual, practical activities, practice, refresher trainings | 9 | "I knew how to prepare a lesson plan before the session. I got skills of talking respectfully." [C]  "The refresher reminds me of the things that I had forgotten from the training." [A] | "Where content was supported by a swap staff [in their language] then it was easier to deliver." [1]  "Mostly in the community people understand when we do it practically so I would say the training was good. The training was- almost 90 percent of it was practicals [demonstration and practice]." [2]  "The CHVs had a curriculum and it was really their guidance. The session guides we had were very important because it was a very useful tool for the CHVs to be able to correctly deliver the sessions to the mothers." [2]  "The CHVs were free enough to ask us questions where they don’t understand compared to when they were trained with the sponsors." [1] |
| Unhelpful/poor elements:  Language barriers, too much to learn | 5 | "Maybe it is the sponsors English that scared us." [A]  "When we started it … delivering was a bit hard." [B] | "When they first came there was an issue of accent that even was a problem to some of us. At the beginning, they were talking very fast." [2]  "It was the first time that we were being introduced to this curriculum and we felt that it was a lot …. Everything was new from the materials, what to prepare and then it was also done in one week of which we felt it was too much for us." [2] |
| ***What extra work did you have to do on your own to prepare for sessions?*** | | | |
| Extra preparation required (mention two or more)  - read manual, practice at home, collect materials | 11 | "I have to go through my notes and through the book [Manual]." [B]  " I tried practicing with my children." [C]  "I looked for the play items to make sure I have all of them in the bag." [A] | "Unless the CHV practice that then you would realize that the CHV would fumble in the session. But I kept on encouraging them to go through the script over and over again before they went into a session." [1] |
| ***How many sessions were supervised? How was feedback?*** | | | |
| Number of group sessions supervised. | 11 | All of them. | All of them. |
| Feedback was helpful | 11 | "At first I was not preparing the sessions well. So they could tell me that 'you have skipped this'. I could know what to do when they told me what I had skipped because they were telling me on the spot. They were very good people and I really appreciated because they were correcting me where necessary.” [C] | "When a CHV missed something then I would just go and tell her before we start the practical. So I would say that 'there is this thing that I would like us to do again before we continue with the practical'. So I just show the mothers how we are supposed to do the game." [1] |
| ***How did you [CHV] demonstrate responsive talk to the parents?*** | | | |
| Three correct actions mentioned that included; e.g., let child point/talk to show interest; let child ask question; parent provides verbal elaboration, parent answers question | 8 | “I would tell them to present the picture to the child. The child will point at the picture, so once the child points at it I will talk to the child. So the child will point at the picture as I talk to the child about the picture. I narrate a story to satisfy the child’s curiosity. I demonstrate that. Then I let alone the mothers to do it and I do coaching." [B] | NA |
| ***How easy or difficult was it for CHV to coach parents on responsive talk with the book?*** | | | |
| Offered at least one reason why easy: CHV trained, Mother & Child loved book, etc. | 11 | "They were so interested in what I was teaching them. I saw the children really loved reading this book. When they saw us opening the book the children would start saying, “mother, socks, father” so that made it easier for me." [A] | "We told the CHVs to tell the mothers to use the language that the child understands." [1] |
| Offered at least one reason why hard: book torn, Mother can’t read, Mother wants to read, etc. | 8 | "The mothers were told to let the child to point on the picture; some mothers were dictating on what the child should say. So it was hard since mothers love commanding their children. At the end of it the child is the one to point." [C] | "When they brought the books, we realized that some of these words are things that the children can’t relate with, probably because the words were too big." [1] |
| ***How did you [CHV] demonstrate responsive play to the parents?*** | | | |
| Named three correct actions that included; e.g., let child pick the game, let child take the lead, parent follows, take turns, praise child's play, raise or lower difficulty level according to ability. | 0 | “If it is the stick play I would take again the child and sit with him/her then I start by giving the baby the stick then I would place the stick and the child would do the same. Then I would take another stick and give it to the baby because the intention is to make a pattern; then I would tell the child how to place it. Even when the baby does not place it well I still encourage her and this makes her happy.” [C] | NA |
| ***How easy or difficult was it for CHV to demonstrate games to the parents?*** | | | |
| Offered one reason why easy: free materials, CHV trained, children love it | 10 | "It was very easy for me because I had the manual and I had been trained and also after training we could also do some refresher courses. So when I go to a session I could take one child and explain with the pictures. The children had gotten used to me because they were seeing me every session and also at their homes." [A] | " they got used to the program and understood what was expected of them… during the refresher training this was addressed, and after that from the practice, we could tell that at least now the CHVs are able to master them [2]  "For the sticks [game] it was easy for them because the sticks are available, the mothers carried their own for the session." [1] |
| Offered one reason why difficult: some parents refuse to play, hard for infants | 6 | "Those puppets were scaring some of the children but at long last they started enjoying" [C] | "I would say there was a lot of confusion—you see like the game of sticks, we were starting with the simpler way of doing it. But when we go to a real scenario, you find that the CHV is not beginning from the simpler stages of the game." [1] |
| ***How easy or difficult was it for you to facilitate open discussion about challenges to practices?*** | | | |
| Offered one or more reasons why easy: over time became comfortable | 10 | "Because they could open the discussion and then they get answers by themselves. Like for example someone can say that 'I cannot manage to do this because of poverty.' So as the CHV I was not answering them but I could leave the floor for them to discuss on that then I will just conclude at the end. Because you know when you give your opinion, then it will not be right because they will say that it was your idea." [C] | "We had refreshers before the session starts. So during these times we try to explore the possible challenges based on each question. So we were just exploring so that if they go back in their groups then they are comfortable enough to handle it." [1] |
| Offered one reason why difficult: some Mothers shy, some dominate, no confidentiality | 6 | “At first it was not easy because even me I could not do it the right way. I also had challenges teaching them because I was also fumbling with whatever I was doing, later on I became familiar with the activities and I also think that we had started getting used to one another. I could now ask questions and they answer or sometimes they could share the challenges that they faced and we could discuss together without fear." [B] | "At first some of the CHVs were a bit hesitant and a bit reserved." [1]  "At times they could even forget about some of the key issues like how to harmonize, because in a discussion I will get a wrong answer, a good answer or a better answer, but I don’t need to refute any answer in a group." [1] |

Note. Frequency for CHVs refers to the number out of 11 who offered a codeable answer. Frequencies are not provided for supervisor FGDs because there were only two.

### Supplementary Table 2. Caregiver Enactment of Program Practices, Barriers, Enablers – Mother and CHV perspectives

|  | **Mothers (N=8 pairs)** | | **CHVs (N=11) comments on mothers** | |
| --- | --- | --- | --- | --- |
| ***Key practice message/frequency of interviewees responding*** | **Quotes** | | **Quotes** | |
|  | **Difficult** | **Easy** | **Difficult** | **Easy** |
| Food message | "Sometimes there was not enough money to buy the foods that the child needed." [B] | "What made it easy for me is that I had a kitchen garden and I could take some and sell to buy eggs and fish for the child." [A] | "Sometimes they did not even have money to buy sardines." [B] | "They reared chickens who gave them eggs." [B] |
| *frequency* | 6 | 7 | 5 | 7 |
| WASH message | "You know when a child is hungry; the child will just rush and pick food without washing the hands." [B] | "I have a place for handwashing and my family knows that they must wash their hands after visiting the toilet and also before feeding." [B] | "At first they said they did not have time to wash." [C] | "If they don't have soap, they use ash." [C] |
| *frequency* | 2 | 4 | 1 | 8 |
| Play message | “Initially we thought toys were not available. I was asking myself, 'how can I leave my chores to go and play with the child?' " [C] | "Program gave me practical lessons on games. I will give the child water and a few cloths to play with. So I will continue with my chores as the child plays." [C] | "They complained that they could not find 15 minutes to play with the child." [B] "It was difficult to add new things to the playbag." [A] | "I found parents with the child using a stick to play." [B]  "The children were so much engaged when playing with the sticks. Sometimes the children would make their own patterns" [C] |
| *frequency* | 5 | 8 | 8 | 9 |
| Talk message | "Actually there were difficulties in the beginning but …we realized it was not nonsense we were doing." [C] | "[The child] will just go on top of the cupboard and get the book and bring it so that we can read it together with him." [B] | "Mothers did not know that you can talk to a baby who has not yet started talking." [C]  "Some mothers were dictating on what the children should say. So it was hard since mothers love commanding their children. At the end of it the child is the one to point." [C] | "The picture was very helpful." [C]  "Children would be pulling the picture so that they could see it." [C] |
| *frequency* | 1 | 4 | 7 | 7 |
| Love & Respect | [no one said it was difficult or provided a reason] | "They were teaching me to show love to my family by loving myself first." [A] | "At first they found it difficult to talk to their spouse." [B] | "As we progressed through the program, they noted that what they talked about was good." [B] |
| *frequency* | 0 | 2 | 1 | 4 |
| ***Specific practices*** |  |  |  |  |
| Had a playbag for child | "Initially it was very difficult." [C] | "I tried to make a playbag, and here it is!" [A]  "The play bag is just put in a place where the child can find it and so when I am not there the child just pick it and play with the toys." [A] | "Some of them would say that they did not have the time to make those playbags. They did not know that the bags would be good." [A] | "Most of them came to see the importance of the play bag. During the session if I just show the play objects on the ground, the children would pick them up and start playing with them." [C] |
| *frequency* | 8 of 8 mother pairs affirmed | | 9 of 11 CHVs report >50% mothers enacted | |
| Added new things to playbag recently | "The child can even destroy the toys given to him previously." [B]  "After making many playing tools some get broken. Then I have to fix them and add new ones." [B] | "I change the toys from time to time." [C] "We were told that we can make play items from anything and we don't need to buy from the shops." [B] | "Sometimes when you ask them they will tell you that they forgot to add." [B] | "Some added bottles or cloth and the other women would see this. So you would see the others also trying to come with them slowly." [A] |
| *frequency* | 7 of 8 mother pairs affirmed | | 6 of 11 CHVs report >50% mothers enacted | |
| Looked at the book daily | "She [child] doesn’t let anybody touch the book." [B] | "Now I can see the child enjoying reading the book and looking at the pictures." [B] | [no CHV commented on a difficulty] | "They tell me the child loves the book and won't put it down." [A] |
| *frequency* | 6 of 8 mother pairs affirmed | | 11 of 11 CHVs report >50% mothers enacted | |

Note. Frequencies refer to the number of mother pairs or CHVs who offered a codeable comment on the question. Mothers were probed about what made it difficult and easy, so frequencies of mother pairs providing a reason are tabulated. CHVs estimated mothers' practices, but not reasons. Quotes from 8 pairs of mothers and 11 CHV interviews are identified by sub-county as A, B, C.
